# Early Single-Center Experience of DaVinci^®^ Single-Port (SP) Robotic Surgery in Colorectal Patients

**DOI:** 10.3390/jcm13102989

**Published:** 2024-05-19

**Authors:** Hye Jung Cho, Woo Ram Kim

**Affiliations:** Division of Colorectal Surgery, Department of Surgery, Gangnam Severance Hospital, Yonsei University College of Medicine, 211 Eonju-ro, Gangnam-gu, Seoul 06273, Republic of Korea; hyejung9025@yuhs.ac

**Keywords:** colorectal neoplasm, robotic surgery, single-port

## Abstract

**Background:** DaVinci^®^ single-port (SP) robotic surgery offers several benefits compared to traditional multiport laparoscopic or robotic surgeries. One of the main advantages is that it allows for a minimally invasive approach, resulting in a single, smaller incision and reduced trauma to the patient’s body, leading to less postoperative pain, faster recovery, and reduced risk of complications. The cosmesis of a single port with minimal visible scarring is also an attractive aspect to the patients; however, many surgeons use an additional port for energy device, stapler use, and drain insertion. Pure single-port surgery with one incision is still rare. Here, we share our experience of our first 10 cases using the SP robotic platform in colorectal surgery. **Methods:** From May 2023 to December 2023, colorectal patients who underwent SP robotic surgery were analyzed. Placement of the incision was the umbilicus for eight patients, and right lower quadrant for two patients, through which ileostomy maturation was performed. Data on perioperative parameters and postoperative outcomes were analyzed, with a median follow-up of 4.6 months (range 0.6–7.4 months). **Results:** A total of 10 colorectal patients underwent DaVinci^®^ single-port robotic colorectal surgery at our institution during this period. The patient demographic was four males (40%) and six females (60%) with a median age of 63.5 years (range 50–75 years). Median body mass index (BMI) was 22.89 kg/m^2^ (range 19.92–26.84 kg/m^2^). Nine patients were diagnosed with colorectal cancer, and one patient was diagnosed with a rectal gastrointestinal tumor. One patient underwent anterior resection and cholecystectomy simultaneously. Mean operation time was 222 min (range 142–316 min), and mean wound size was 3.25 cm (range 2.5–4.5 cm). Nine patients underwent surgery with single incision through which a single-port trocar was inserted, and one patient had one additional port for drain insertion. Mean hospital stay was 6 days (range 4–8 days) with one postoperative complication of bleeding requiring transfusion, but there was no readmission within 30 days. **Conclusions:** Overall, our experience with single-port robotic colorectal surgery has been promising. With only one patient with additional port for drain insertion, all nine patients underwent SP-robotic surgery with single incision for colon as well as rectal surgeries. Compared to an average postoperative length of stay of 6.5-8 days in laparoscopic colorectal surgeries reported in literature, SP-robotic surgery 33showed faster recovery of 6 days highlighting its benefits in patient recovery and satisfaction.

## 1. Introduction

Minimally invasive surgery (MIS) has been at the center of the surgical field since the 1980s. Over the last four decades, surgical innovations have taken an enormous stride into the robotic platform. With its introduction in 2002, multiport robotic surgery gained popularity, offering enhanced precision and stability, dexterity with better ergonomics, and three-dimensional visualization [1]. A comparative study between laparoscopic and robotic colorectal surgery showed comparable results in postoperative incisional hernias, anastomotic leakage, surgical site infections, and open conversion rates [2]. A review article comparing laparoscopic versus robotic colectomies showed shorter length of hospital stay, lower rate of open conversion, and lower intraoperative complication rate for the latter [3]. Robotic approaches even showed improved long-term surgical outcomes with comparable lymph node retrieval and negative surgical margins in a study carried out by Mirkin et al., laying the ground for robotic colectomy as one of the standard surgical procedures for colorectal surgeons [4].

With further advancement, DaVinci^®^ single-port robotic surgery (SPRS, Intuitive Surgical, Sunnyvale, CA, USA) is the utmost cutting-edge system designed to enhance the performance of complex surgeries with minimal invasiveness. In addition to the benefits of ergonomics provided by the robotic system, SPRS provides additional benefits of cosmesis, reduced postoperative pain requiring analgesics, and reduced costs [5,6,7]. With its clinical debut in 2018, the SPRS platform has been mainly used in urology, gynecology, and general surgery, such as cholecystectomy and adrenalectomy [8]. However, its application in colorectal surgery was dormant.

With its first report in 2020 by Marks et al., SPRS for colectomy has gained momentum in many colorectal surgeries [9]. Comparative studies between SPRS and single-incision laparoscopic surgery (SILS) have reported similar operation time, complication rates, and pathological outcomes, confirming its safe and feasible use [10,11]. The hesitant application of SPRS in colorectal surgery pertains to rectal surgery due to the absence of stapling device and angulation.

Despite the apparent benefits of SPRS, there remains a significant gap in research regarding long-term outcomes, patient selection criteria, and technical optimization. As SPRS is currently approved for clinical use in South Korea, Japan, and the United States with limited FDA approval, colorectal surgeries using SPRS are still in the early stages with limited sample size. Further studies are needed to clarify the full potential of SPRS and address its limitations. The authors appreciate the importance of sharing our experience and shedding light on ways to overcome some of the difficulties with the SPRS.

## 2. Materials and Methods

### 2.1. Patient Selection

Data from patients who underwent colorectal surgery using the DaVinci single-port (SP) robotic platform between May 2023 and December 2023 at the Gangnam Severance Hospital, Yonsei University College of Medicine (Seoul, Republic of Korea) were reviewed. Patients in need of surgery due to colorectal cancer were given the option of laparoscopy or robotic surgery. Due to the national health insurance policy, patients with private insurance opted for robotic surgery, while those without preferred the laparoscopic method. A total of 10 consecutive patients who underwent SPRS were analyzed.

### 2.2. Surgical Procedures

**Figure 1:**
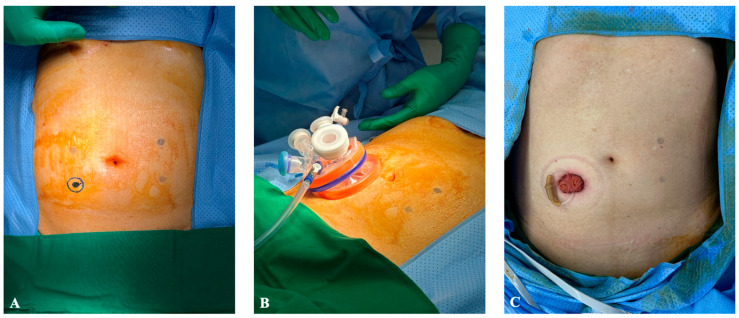
Port placement for ultra-low anterior resection. (**A**) Circular incision at right lower quadrant. (**B**) Four-chamber glove port inserted. (**C**) Loop ileostomy maturation at right lower quadrant incision site.

**Figure 2:**
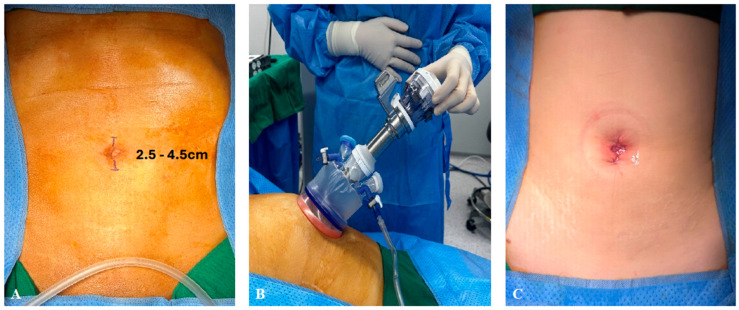
Port placement for colectomy using transumbilical incision. (**A**) Transumbilical incision (range 2.5–4.5 cm) for glove port insertion. (**B**) Four-chamber glove port inserted. (**C**) Immediate postoperative transumbilical wound.

For right-sided colectomy, the patient lied in a supine position. A transumbilical incision was made through which a 4-chamber SP glove port (Meditech Inframed, Seoul, Republic of Korea) was inserted. For left-sided colectomy, the patient lay in a position for lithotomy. Similarly, a transumbilical incision was made for the glove port; however, for two ultra-low anterior resections, a circular incision was made at the right lower quadrant at the position of ileostomy maturation. Incisions can be visualized in Figure 1 and Figure 2. The SP robotic arms were positioned and docked from the ipsilateral side of the lesion by the assistant, and the docking time was less than 3 min in all cases.

There was one case of an additional port inserted in the right lower quadrant for low anterior resection. A laparoscopic stapler (Signia^TM^, Medtronic, Minneapolis, MN, USA) was inserted for distal margin resection and drain insertion. The other 9 cases were performed with a single incision.

For fascia closure, a barbed suture (Stratafix^TM^, Ethicon Inc., Cincinnati, OH, USA) was used with an absorbable, synthetic, barbed suture (3-0 vicryl*, Ethicon Inc., USA) and skin adhesive (Liquiband^®^, Advanced Medical Solutions Ltd., Lenexa, KS, USA).

### 2.3. Measurement of Clinical Variables

Demographics of the patients including age, sex, American Society of Anesthesiologists (ASA) classification, and body mass index (BMI) were collected. Clinical data reviewed included the diagnosis of the patient for which surgery was performed, operation date, and discharge date. Length of hospital stay (days) was defined as day of surgery to discharge date. Intraoperative results, such as operation time (min), wound size (cm), blood loss (mL), transfusion status, ileostomy status, wound location, and drain insertion status, were analyzed. Patients graded the pain level using a numeric pain rating scale (NPRS, 1–10) daily, and NPRS scores on postoperative days 1 and 3 were reviewed. The length of intravenous (IV) pain medication was also collected. After discharge, pathological data on tumor size, TNM stage, lymphovascular (LVI) and perineural invasion (PNI) status, and adjuvant chemotherapy status were reviewed at the outpatient clinic. Postoperative complications graded according to Clavien–Dindo classification were assessed. Median follow-up was 6.6 months (range 2.6–9.4 months).

### 2.4. Statistical Analysis

All analyses were performed using R version 4.2.1 (R Foundation for Statistical Computing, Vienna, Austria, https://www.r-project.org/, accessed on 1 March 2024). Data on perioperative parameters and postoperative outcomes were analyzed. Continuous variables were analyzed using t-tests and are presented as means, medians, and standard deviations with a range. Categorical variables were analyzed using the chi-squared test or Fisher’s exact test and are presented as frequencies.

## 3. Results

Data from 10 patients (4 male (40%), 6 female (60%); mean (±SD) age, 62.9 ± 9.6 years) were analyzed. Mean BMI was 22.87 ± 2.5, and ASA status varied (ASA 1, 1 (10%); ASA 2, 4 (40%); ASA 3, 5 (50%)). The diagnosis of the patients was divided into nine (90%) colorectal cancer and one (10%) rectal gastrointestinal tumor. The surgical procedure was carried out according to the location of the lesion, and comprised two right hemicolectomies, five anterior resections, one low anterior resection, and two ultra-low anterior resections. One patient had double primary cancers at the descending and sigmoid colon for which anterior resection was performed, and one patient underwent cholecystectomy in addition to anterior resection for primary sigmoid colon cancer. The baseline demographics of the patients are shown in Table 1.

Mean operation time was 222.4 ± 66.1 min (range 142–316 min) with a mean blood loss of 148 ± 126.2 mL (range 50–400 mL). In two patients for whom ultra-low anterior resection was performed, a single-port incision was made at the right lower quadrant. Ileostomy was maturated for both patients at the end of the surgery through the single-port site. (Figure 1C). Transumbilical incisions were made for the remaining eight patients, and the mean wound size was measured at 3.25 ± 0.6 cm (range 2.5–4.5 cm) (Figure 2). No intraoperative blood transfusion was given. The average tumor size was 3.15 ± 1.9 cm (range 1.2–6.9). Four patients underwent adjuvant chemotherapy, and the pathological stages of the patients are shown in Table 2.

Median NPRS score on postoperative days 1 and 3 was 3 (range 2–3) on both days. No patients required IV pain medication after 2 days. Mean hospital stay was 6 ± 1.2 days (range 4–8 days), and no readmission within 30 days was noted. One patient had a grade II complication for which blood transfusion was required for low hemoglobin level (6.9 g/dL). The patient was discharged on postoperative day 5 without any sequelae. These results are shown in Table 3.

## 4. Discussion

Our exploration into the application of the SPRS system for colorectal procedures has illuminated several critical insights. The system’s integration into colorectal surgery represents a significant advancement in MIS, echoing the broader shift towards robotics in the surgical field. The utilization of SPRS in colorectal surgery, while initially hesitant, has seen a burgeoning interest following reports of its successful application in colectomy.

The ergonomics of the robotic platform are an undisputable advantage compared to the laparoscopic platform [12]. Eisenberg et al. reported significantly shorter suturing time with the robotic system than with standard laparo-endoscopic single-site surgery (*p* < 0.0001) [13]. The risk of bypassing drain insertion, especially for low anterior resection, may hold some risk. However, intracorporeal reinforcing suturing of the anastomosis using the robotic system can reduce the possible occurrence of anastomosis leakage. A recent systematic review and meta-analysis by Wang et al. demonstrated a lower incidence of anastomosis leakage (RR 0.41, 95% CI 0.25–0.66) in patients with reinforcing sutures, with a leakage rate of 4.4% compared to the 11.9% for whom no reinforcing suture was performed [14].

Our data align with the existing literature that compares SPRS with single-incision laparoscopic surgery (SILS), showing comparable operation times [6,7]. Our mean operation time of 222.4 min is longer than that reported by Change et al. (185 min), but shorter than in the comparative analysis performed by Keller et al. (296 min). Anastomosis methods were identical to laparoscopic methods, ensuring similar anastomosis time required. However, we performed one case of reinforcing suture in low anterior resection after anastomosis using a circular stapler. We had one case of a grade II Clavien–Dindo complication of postoperative anemia (Hg 6.9 g/dL) requiring transfusion. However, the initial hemoglobin level of the patient was low (8.5 g/dL), with hematocrit of 28.1% (normal range 37–47%). The patient was discharged without any sequelae on postoperative day 5. There were no readmissions within 30 days.

The mean wound size of 3.25 cm was comparable to all other studies regarding SPRS colectomy. Lim et al. reported a transumbilical incision length of 4–6 cm, and Bae et al. reported 5.0 cm [15,16]. Initial experience by Marks et al. reported an incision length of 4.0 and 4.5 cm in SPRS [9]. Our transumbilical wound incision length of 3.25 cm is one of the shortest incision lengths yet reported, emphasizing the cosmetic benefit of SPRS.

The comparative analysis of length of hospital stays, postoperative pain levels, and the limited need for IV pain medication post-surgery attests to the SP system’s advantage in enhancing patient recovery experiences. Mean length of hospital stay was 6 days, comparable to the 2–9 days reported by a recent systemic review of SPRS for colonic disease [17]. Forty percent (4/10) of patients did not require any IV analgesic after the day of operation. Two patients required IV pain control up to postoperative day 1, and the rest (40%) up to postoperative day 2. These findings coincide with the overarching goals of MIS to reduce hospitalization duration and expedite patient recovery, pivotal in the current health-care landscape focused on patient-centered outcomes and cost efficiency. The median follow-up of 6.6 months, although limited, provides an initial glance at the postoperative trajectory, with outcomes suggesting a positive recovery profile.

The SP system has some drawbacks. Due to the absence of an SP-synced stapler, an additional trocar is often inserted. The resection of the distal margin in upper-to-middle rectal cancer using the laparoscopic stapling device using the same single-port trocar site may be difficult due to the angle of the device and the narrow space of the pelvic cavity. Many of the studies reporting single-incision robotic colectomies often used an additional port for such a reason [15,18,19]. In our study, two patients had drain insertions. The first patient was our first colectomy using the SP robotic platform, and the drain was inserted through the umbilical incision site. The second patient was diagnosed with rectal cancer and a low anterior resection was performed. An additional port was inserted in the right lower quadrant, through which the assistant used a laparoscopic stapler for distal margin resection, and a drain was inserted through the additional port at the end of the surgery. Although the recent rise in the use of ERAS (enhanced recovery after surgery) protocol strongly recommends no drainage of the peritoneal cavity or pelvis after colorectal surgery, the role of early detection of anastomosis leakage hinders many surgeons from relinquishing the procedure [20]. In order to compensate for such a burden, for patients with upper-to-middle rectal cancer for whom low anterior resection is required, a drain may be inserted through the additional port. Based on our experience, we suggest that optimal indications for true SPRS may include early-stage colorectal cancers located from the cecum to the rectosigmoid junction. This range facilitates the use of a stapler for resection through the single-port site. Furthermore, for cases of low rectal cancer where a diverting ileostomy is necessary, an incision in the right lower quadrant may represent an additional appropriate indication for SPRS.

The most remarkable benefit of SPRS is the ability to perform multi-quadrant operation with a single incision. To our knowledge, other than a case report shared by Juo et al., no other attempts have been reported on performing subtotal or total colectomy using a single incision [21]. Patients with inflammatory bowel disease (IBD), such as ulcerative colitis requiring total proctocolectomy, may benefit from a single incision, and the advancement of IBD surgery is open for advancement with the SPRS once doors open with FDA approval in the US as well as adoption of SPRS in Europe this year [22].

Despite these promising results, our study had several limitations, the first of which was the inherent constraints of its retrospective design. The relatively small sample may also constrain the generalizability of the findings. To date, all reported findings regarding colectomy using SPRS have been from case studies. Future research should aim to expand on these preliminary insights through multicenter trials and larger patient cohorts, exploring long-term outcomes and further delineating the criteria for patient selection to optimize benefits.

Overall, our experience with single-port robotic colorectal surgery has been promising. With only one patient with an additional port for drain insertion, all nine patients underwent SP robotic surgery with a single incision for colon as well as rectal surgeries. Compared to an average postoperative length of stay of 6.5–8 days in laparoscopic colorectal surgeries reported in the literature, SP robotic surgery showed faster recovery of 6 days, highlighting its benefits in patient recovery and satisfaction.

## 5. Conclusions

Overall, our experience with single-port robotic colorectal surgery has been promising. With only one patient with an additional port for drain insertion, all nine patients underwent SP robotic surgery with a single incision for colon as well as rectal surgeries. Compared to an average postoperative length of stay of 6.5–8 days in laparoscopic colorectal surgeries reported in the literature, SP robotic surgery showed faster recovery of 6 days, highlighting its benefits in patient recovery and satisfaction. While still in its early stages, SPRS in colorectal surgeries is safe and feasible, with benefits in cosmesis and perioperative outcomes. Further improvements can be made once single-port compatible energy and a stapling device is introduced. Future studies on long-term oncologic outcomes are warranted.

## Figures and Tables

**Table 1:** Baseline characteristics of included patients.

Patient No.	Age (Years)	Sex	BMI (kg/m^2^)	Diagnosis
1	67	M	23.65	Rectosigmoid junction cancer
2	60	F	21.10	Sigmoid colon cancer
3	69	F	22.38	Ascending colon cancer
4	50	F	20.23	Rectal cancer
5	71	F	26.84	Rectal cancer
6	56	M	23.92	Descending + sigmoid colon cancer
7	52	F	20.63	Splenic flexure cancer
8	75	M	19.92	Ascending colon cancer
9	75	M	26.60	Rectal GIST
10	43	F	23.40	Sigmoid colon cancer + cholecystitis

GIST, gastrointestinal stromal tumor; BMI, body mass index.

**Table 2:** Perioperative and histologic outcome.

Patient No.	Operation Time (min)	Blood Loss (mL)	Wound Incision	Wound Size (cm)	Tumor Size (cm)	Pathological Stage	Adjuvant Chemotherapy Status
1	220	50	Umbilical	4.5	5.5	IIIB	Yes
2	151	50	Umbilical	4	1.2	I	No
3	262	100	Umbilical	3.5	6.9	IIA	Yes
4	312	350	Right lower quadrant	3	3.7	I	No
5	275	100	Umbilical	3.5	1.4	IIIA	Yes
6	142	100	Umbilical	2.5	2.4	I	No
7	190	50	Umbilical	2.5	1.8	I	No
8	207	100	Umbilical	3	3.1	IIA	Yes
9	316	400	Right lower quadrant	3	4.1	-	No
10	149	180	Umbilical	3	1.4	0	No

**Table 3:** Postoperative outcome.

Patient No.	Pain Score, POD#1 (NPRS)	Pain Score, POD#3 (NPRS)	IV Pain Medication (Days)	Hospital Stay (Days)	Postoperative Complication (Clavien–Dindo Classification)
1	2	3	0	6	
2	3	3	0	7	
3	3	2	0	5	
4	3	3	1	5	II, postoperative anemia
5	7	3	1	8	
6	5	3	2	4	
7	3	2	2	6	
8	3	2	2	5	
9	3	2	0	7	
10	2	3	2	7	

NPRS, numeric pain rating scale; IV, intravenous.

## Data Availability

The datasets generated and analyzed during the current study are available from the corresponding author upon reasonable request.
